# Signal Associa: hot tumors elicit field-spanning NK cell coordination linked to their recruitment

**DOI:** 10.64898/2026.10.21.760734

**Published:** 2026-10-23

**Authors:** Majid Rostami, Hyeri Choi, Yeonwoo Rho, Noo Li Jeon, Sangyoon J. Han

## Abstract

Cells coordinate through coupled dynamics, yet whether observed correlations reflect genuine coupling is rarely tested. Signal Associa is an open-source framework uniting six coupling methods with automated stationarization and surrogate testing. Benchmarking against six ground truth systems reveals a trichotomy of coupling regimes, each with a different best method: correlation for coupling carried by shared trends and cycles, Granger causality for linear stochastic coupling, and convergent cross-mapping for nonlinear coupling; agreement across methods identifies the regime from data. In 13 movies of natural killer (NK) cells across three colorectal tumor microenvironments, NK cells formed field-spanning coupling networks at a conserved rate; at matched exposure to tumor signal, NK cells around hot tumors were more globally integrated, and hot tumors with a more integrated NK network tended to attract more NK cells. In focal adhesions, stiffness strengthened vinculin–FAK coupling, most at protruding edges. Surrogates cut lagged-correlation false positives from 23–92% to about 5%.

